# LncRNA HOXA-AS3 promotes gastric cancer progression by regulating miR-29a-3p/LTβR and activating NF-κB signaling

**DOI:** 10.1186/s12935-021-01827-w

**Published:** 2021-02-18

**Authors:** Feng Qu, Bin Zhu, Yi-Lin Hu, Qin-Sheng Mao, Ying Feng

**Affiliations:** 1 Department of Gastrointestinal Surgery, Affiliated Hospital of Nantong University, Nantong, China; 2 Department of General Surgery, Rudong Third People’s Hospital, Rudong, China; 3 Research Center of Clinical Medicine, Affiliated Hospital of Nantong University, Nantong, China

**Keywords:** HOXA-AS3, Gastric cancer, miR-29a-3p, Ltβr, NF-κb

## Abstract

**Background:**

Gastric cancer (GC) is among the most common and deadliest cancers globally. Many long non-coding RNAs (lncRNAs) are key regulators of GC pathogenesis. This study aimed to define the role of HOXA-AS3 in this oncogenic context.

**Methods:**

Levels of HOXA-AS3 expression in GC were quantified via qPCR. The effects of HOXA-AS3 knockdown on GC cells function were evaluated in vitro using colony formation assays, wound healing assays and transwell assays. Subcutaneous xenograft and tail vein injection tumor model systems were generated in nude mice to assess the effects of this lncRNA in vivo. The localization of HOXA-AS3 within cells was confirmed by subcellular fractionation, and predicted microRNA (miRNA) targets of this lncRNA and its ability to modulate downstream NF-κB signaling in GC cells were evaluated via luciferase-reporter assays, immunofluorescent staining, and western blotting.

**Results:**

GC cells and tissues exhibited significant HOXA-AS3 upregulation (*P* < 0.05), and the levels of this lncRNA were found to be correlated with tumor size, lymph node status, invasion depth, and *Helicobacter pylori* infection status. Knocking down HOXA-AS3 disrupted GC cell proliferation, migration, and invasion in vitro and tumor metastasis in vivo*.* At a mechanistic level, we found that HOXA-AS3 was able to sequester miR-29a-3p, thereby regulating the expression of LTβR and modulating NF-κB signaling in GC.

**Conclusion:**

HOXA-AS3/miR-29a-3p/LTβR/NF-κB regulatory axis contributes to the progression of GC, thereby offering novel target for the prognosis and treatment of GC.

## Introduction

Gastric cancer (GC) was the 5th most prevalent form of cancer diagnosed in 2018 [1]. While GC mortality rates have been declining over the past five decades, it is still the third leading cancer-associated cause of death [2]. GC patients often have a poor prognosis because they are typically diagnosed when the disease is in an advanced stage and because GC exhibits high rates of chemoresistance. It is thus essential that novel biomarkers and therapeutic targets associated with GC be identified so as to facilitate the diagnosis and treatment of affected patients.

Long non-coding RNAs (lncRNAs) lack the ability to encode protein, but are capable of regulating cellular physiology through complex epigenetic mechanism [3]. Many functionally important lncRNAs have been detected and studied in recent years, and have been shown to suppress or promote GC invasion, growth, and metastasis [4, 5].

HOXA cluster antisense RNA 3 (HOXA-AS3) is a 3992 nucleotide lncRNA encoded on chromosome 7p15.2 in the HOX gene cluster, which encodes highly conserved and homologous transcription factors which play key roles in the process of embryo development [6], in addition to controlling hematopoietic cell differentiation [7]. HOXA-AS3 expression has previously been linked to poor prognosis in patients with hepatocellular carcinoma (HCC) and glioma [8, 9], and at a functional level, it has been shown to promote the proliferative and migratory activity of HCC, glioma, and lung adenocarcinoma cells [8–10]. There is also evidence that HOXA-AS3 can mediate cisplatin resistance, making it a viable therapeutic target in non-small cell lung cancer (NSCLC) [11]. HOSX-AS3 has also been confirmed to serve as a competing endogenous RNA (ceRNA) in HCC and glioma [9, 12], whereas its functional role in GC remains to be described.

Herein, we clarified a novel oncogenic role for HOXA‐AS3 in human GC. Knocking down this lncRNA disrupted its ability to function as a ceRNA for miR-29a-3p, thus resulting in the downregulation of LTβR, thereby altering NF-κB pathway activation and disrupting GC cell proliferation, invasion, and migration. Together, these data suggest that HOXA-AS3 may thus be an important clinical target in GC for the treatment or prevention of this disease.

## Methods

### Patients and tissue samples

82 pairs of GC tissues and matched adjacent normal tissues were collected from patients who underwent standard D2 lymphadenectomy from March to September 2010 at Department of Gastrointestinal Surgery of Affiliated Hospital of Nantong University. All patients with clear GC pathology did not harbor detectable distant metastasis or malignant tumors in other sites upon preoperative examination, and they were not administered neoadjuvant chemotherapy, radiotherapy, immunotherapy or other specialized treatment. Immediately after collection, the tissues were snap-frozen in liquid nitrogen and stored in – 80℃ until further use. Both GC samples and normal tissues were confirmed by pathological examination. Follow-up was completed by September 2015. Overall survival (OS) and disease‐free survival (DFS) refer to the interval from the time of surgery to death or recurrence, respectively. The demographic and clinical characteristics of the patients were shown in Table 1. Complete medical and follow‐up data were available for all patients. Approval was obtained from the Human Research Ethics Committee of Affiliated Hospital of Nantong University, and written informed consent was obtained from each patient (Nos. 2020-L121).

**Table 1:** Relationship between patient characteristics and HOXA-AS3 expression levels in tissues

Clinicopathologic features	n	HOXA-AS3 expression		*χ*^2^	*P* value
		Low	High		
Gender				0.467	0.464
Male	51	27	24		
Female	31	14	17		
Age (years)				0.668	0.414
≤ 50	17	7	10		
> 50	65	34	31		
Tumor diameter (cm)				9.848	0.002**
≤ 4	48	31	17		
> 4	34	10	24		
Tumor location				0.201	0.654
Up + Middle	34	16	18		
Down	48	25	23		
Tumor differentiation				2.390	0.122
High + Middle	41	24	17		
Low	41	17	24		
CEA level (ng/ml)				3.514	0.061
≤ 5	70	38	32		
> 5	12	3	9		
CA 19-9 level (U/ml)				1.242	0.265
≤ 37	66	35	31		
> 37	16	6	10		
HP infection				3.989	0.046*
−	37	23	14		
+	45	18	27		
T stage				20.500	< 0.0001****
T1-2	32	26	6		
T3-4	50	15	35		
N stage				25.866	< 0.0001****
N0	39	31	8		
N1-3	43	10	33		

*P < 0.05; **P < 0.01; ****P < 0.0001

### Cell culture

Human GES-1 control gastric mucosal cells and the MGC-803, AGS, MKN-45, SGC-7901, and HGC-27 GC cell lines were from GeneChem (Shanghai, China) and were grown at 37 °C in a humidified 5% CO_2_ incubator in RPMI-1640 containing 10% fetal bovine serum (FBS) and penicillin/streptomycin.

### qPCR

TRIzol (Invitrogen, USA) was utilized to extract total cell RNA to conduct qPCR as in prior reports [13], with GAPDH and U6 being used as normalization controls. Primers used in this study were from RiboBio (Guangzhou, China). Primer sequences were as follows:

HOXA-AS3 forward: 5′-ACCAAAGATTCCGTTTTCAAGG-3′;

HOXA-AS3 reverse: 5′-TGCCCACAATAGAGTGTATGCC-3′;

LTβR forward: 5′- ATGCGCCCGCCTTGGGCC -3′;

LTβR reverse: 5′- TCAGAGGGAGTGGCAGC -3′

miR-29a-3p forward: 5′-CTGAGTTTCTATTTAGACACTACAACA-3′;

miR-29a-3p reverse: 5′-ACAATTTGACATGTGGCATTAACG-3′;

GAPDH forward: 5′-AGAAGGCTGGGGCTCATTTG-3′;

GAPDH reverse: 5′-AGGGGCCATCCACAGTCTTC-3′.

U6 forward: 5′-AGCGGGAAATCGTGCGTGACA-3′;

U6 reverse: 5′-GTGGACTFGGGAGAGGACTGG-3′

### Cellular transfection and transduction

Three different HOXA-AS3-specific siRNA sequences and a control sequence encoded in lentiviral vectors were purchased from GeneChem and were transduced into MKN-45 and SGC-7901 cells, after which qPCR was used to gauge relative knockdown efficiency. GAPDH was used for normalization purposes, with the 2^−ΔΔCt^ method being used to assess levels of gene expression.

All miR-29a-3p mimic/inhibitor/nc were constructed from GenePharma (Suzhou, China), while pcDNA-3.1/LTβR plasmids and corresponding controls were obtained from GeneChem. Cells were transfected using Lipofectamine 2000 (ThermoFisher Scientific, MA) based on provided directions. At 48 h post-transfection, cells were utilized in downstream assays.

### Proliferation, migration, and invasion assays

Colony formation, wound healing, and transwell assays were conducted as in prior reports [14]. To measure colony formation, we added 200 GC cells per well to a 6‐well plate, followed by culture for 14 days. Cells were fixed with 4% paraformaldehyde, stained with 1% crystal violet, and colonies comprising > 50 cells were counted. For wound healing assays, cells were seeded on 6-well plates, grown to confluency, and scratched with a 200 μL pipette tip. Wound recovery was observed under an IX71 inverted microscope (Olympus) after 0 and 48 h. In transwell assays, cells were seeded on 24-well transwell plates (Corning, New York, NY) to measure their invasive capacity. Inserts were coated with 60 µl of Matrigel (BD Biosciences, Franklin Lakes, NJ). After 24 h, invaded cells were fixed and stained with 0.1% crystal violet solution containing formaldehyde. The numbers of invaded cells were counted in five randomly-selected fields under an IX71 inverted microscope (Olympus).

### Animal experiments

A subcutaneous xenograft tumor model system was generated in nude mice as reported previously [14]. Briefly, SGC-7901 cells were transduced with shNC or shHOXA-AS3 constructs and were implanted subcutaneously in nude mice. Every 5 days, tumor size was monitored, and mice were euthanized after 30 days at which time these tumors were imaged, isolated, and weighed. We additionally established a tail vein injection tumor model system in nude mice [14], injecting these same transformed SGC-7901 cell lines into these animals. Mice were then euthanized 5 weeks later, and metastatic nodules in the lungs were imaged. Hematoxylin and eosin (H&E) staining was conducted to assess the presence of these nodes within the lungs. All experiments were approved by the Animal Care Committee of Nantong University (Nos. S20200323-105).

### Bioinformatics analyses

Levels of miR-29a-3p and HOXA-AS3 expression in GC samples were assessed by querying data downloaded from TCGA (https://gdc.cancer.gov/). The R and GraphPad Prism 8 (GraphPad Software, Inc., CA, USA) software packages were utilized to analyze these data following log2 transformation. In addition, potential miR-29a-3p target genes were detected with the RNA22, mirtarbase, targetscan, and MIRwalk tools, with LTβR being selected as our primary target of interest via Venn diagram (https://creately.com/diagram-type/venn).

### Western blotting

Western blotting was conducted as in prior reports [15], using the following primary antibodies: anti-NF-κB p65 (Cell Signaling Technology, Danvers, MA; 8242, 1:1000), anti-p-IKKβ (Abcam, ab59195, 1:1000), anti-p-IκBα (Cell Signaling Technology, 14D4, 1:1000), anti-LTβR (Abcam, Burlingame, CA; ab65089, 1:1000), anti-GAPDH (Proteintech, Wuhan, China; Cat No. 60004-1-Ig, 1:1000).

### Subcellular fractionation

A PARIS™ Kit (Invitrogen) was utilized to collect fractionated RNA from the nuclei and cytoplasm of GC cells, with U6 and GAPDH being utilized as normalization controls for these two respective fractions.

### Luciferase-reporter assay

Portions of the HOXA-AS3 or LTβR genes containing WT or mutated (MUT) versions of predicted miR-29a-3p binding sites were purchased from GeneChem. The resultant constructs were then co-transfected into SGC-7901 and MKN-45 cells along with miR-29a-3p mimics or control constructs. All Luciferase reporter assays were conducted based on provided protocols, with Renilla luciferase assay kit (Beyotime, Shanghai, China) being utilized as an internal normalization control. Experiments were conducted in triplicate.

### Immunofluorescent staining

SGC-7901 or MKN-45 cells were plated in 24-well plates, fixed, permeabilized, blocked, and incubated overnight at 4 °C with anti-NF-κB p65 (Cell Signaling Technology, 8242, 1:100). They were then rinsed and probed using AF594-linked goat anti-rabbit IgG (ABclonal, Wuhan, China; AS039). DAPI was then used for nuclear counterstaining, and cells were imaged via a BX41 microscope (Olympus).

### Statistical analysis

In the experimental studies data were presented as mean ± SD of more than three independent experiments that were each performed in triplicate. SPSS 24.0 software (IBM SPSS Statistics, IL, USA) was used to analyze data, which were assessed via Student’s t-tests, one-way ANOVAs, or chi-squared tests as appropriate. Survival curves were analyzed using the Kaplan–Meier approach. Factors that were determined to be of prognostic relevance in a univariate Cox regression model were incorporated into a subsequent multivariate Cox regression model. *P* < 0.05 was considered to be the significance threshold for all analyses, and GraphPad Prism 8 was used to prepare all figures.

## Results

### GC tumors exhibit the upregulation of HOXA-AS3, which is correlated with poor patient prognosis

**Fig. 1:**
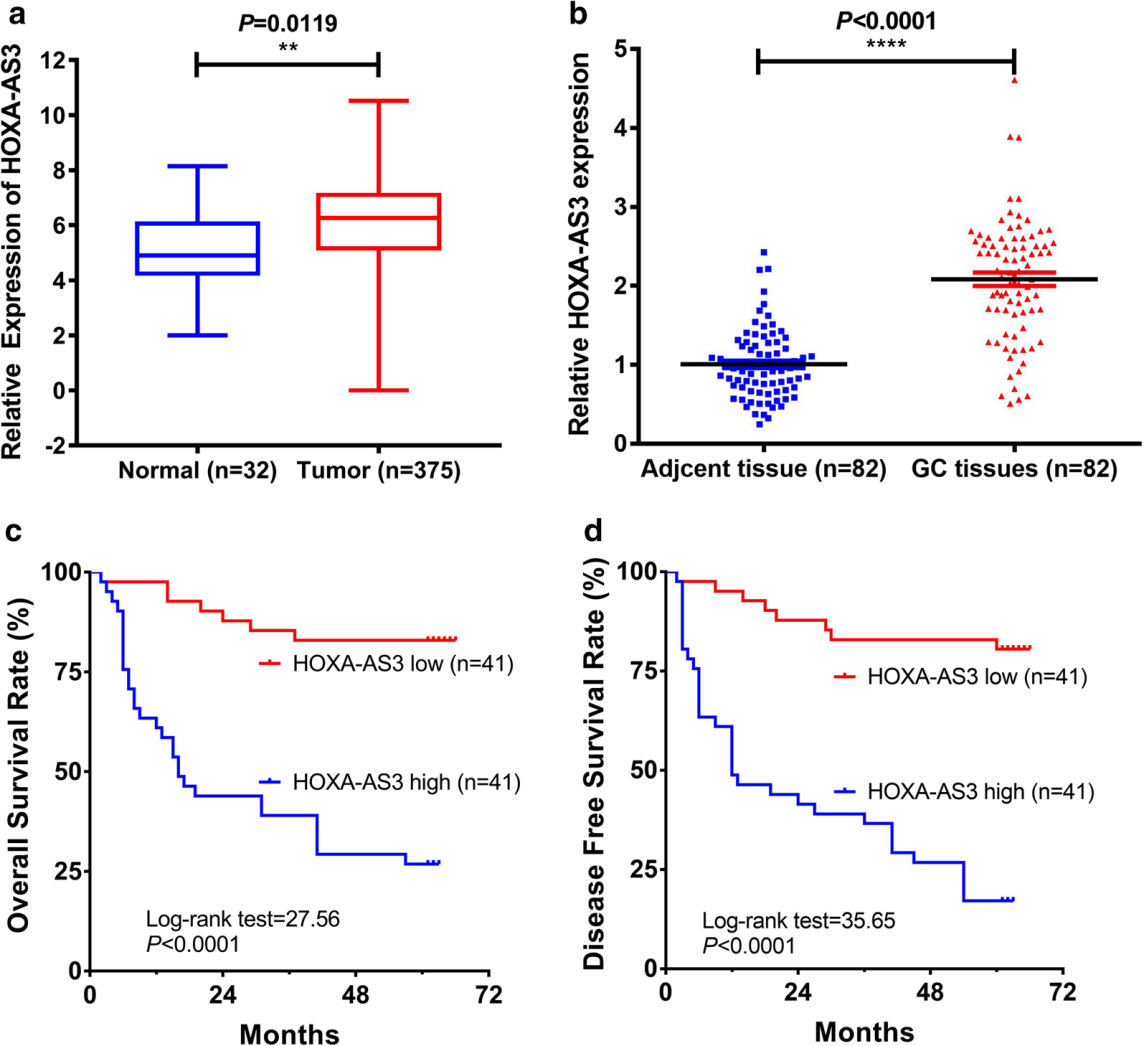
GC patient tumors exhibit HOXA-AS3 upregulation that is correlated with poor patient prognosis. **a** The upregulation of HOXA-AS3 in GC tumor tissues was observed in the TCGA database. **b** Relative HOXA-AS3 levels were measured via qPCR in 82 pairs of GC tumor tissues and paired control samples. **c**,** d** GC patients with high and low levels of HOXA-AS3 expression (n = 41 each) were compared to assess their relative OS and DFS

We began by assessing HOXA-AS3 expression in human GC tissues based upon TCGA microarray data, revealing significant upregulation of this lncRNA in tumor tissues relative to healthy control tissue samples (*P* = 0.0119, Fig. 1a). We further confirmed this finding by using a qPCR approach to analyze 82 pairs of GC tumor and paracancerous tissues (*P* < 0.0001, Fig. 1b), and we then evaluate the relationship between HOXA-AS3 expression levels and clinicopathological findings in these patients. We determined that elevated expression of this lncRNA was correlated with tumor size (*P* = 0.002), T stage (*P* < 0.0001), N stage (*P* < 0.0001), and *Helicobacter pylori* infection status (*P* = 0.046), whereas it was unrelated to gender, age, tumor location, tumor differentiation, CEA levels, or CA 19-9 levels (Table 1). Kaplan–Meier survival analyses revealed that GC patients with higher levels of HOXA-AS3 expression exhibited decreased OS and DFS relative to those expressing lower levels of this lncRNA (both *P* < 0.0001, Fig. 1c, d). Multivariate analyses further revealed that high HOXA-AS3 expression levels were independently predictive of poorer OS and DFS in these GC patients (*P* = 0.003 and *P* = 0.005, Tables 2 and 3).

**Table 2:** Univariate and multivariate analyses of prognostic factors for OS in gastric cancer

Variable	Overall survival			
	Univariate analysis		Multivariate analysis	
	*P* value	HR (95% CI)	*P* value	HR (95% CI)
Age (yr)	0.361	0.705 (0.332–1.494)		
≤ 50 vs > 50				
Gender	0.815	1.084 (0.552–2.130)		
Male vs female				
Tumor diameter (cm)	0.002**	2.804 (1.450–5.422)	0.237	1.585 (0.739–3.399)
≤ 4 vs > 4				
Tumor location	0.719	0.887 (0.463–1.701)		
Up + Middle vs Down				
Tumor differentiation	0.027*	2.125 (1.091–4.137)	0.144	1.681 (0.838–3.375)
High + Middle vs Low				
CEA (ng/ml)	0.386	1.439 (0.632–3.277)		
≤ 5 vs > 5				
CA 19–9 (U/ml)	0.843	0.920 (0.404–2.095)		
≤ 37 vs > 37				
HP infection	0.918	0.967 (0.506–1.846)		
− vs +				
T stage	0.011*	2.645 (1.245–5.618)	0.385	0.640 (0.234–1.752)
T1-2 vs T3-4				
N stage	< 0.0001****	5.800 (2.532–13.288)	0.082	2.377 (0.897–6.300)
N0 vs N1-3				
HOXA-AS3 in tissues	< 0.0001****	6.723 (2.937–15.385)	0.003**	4.662 (1.691–12.856)
High vs Low				

*P < 0.05; **P < 0.01; ****P < 0.0001

**Table 3:** Univariate and multivariate analyses of prognostic factors for DFS in gastric cancer

Variable	DFS			
	Univariate analysis		Multivariate analysis	
	*P* value	HR (95% CI)	*P* value	HR (95% CI)
Age (yr)	0.369	0.722 (0.355–1.470)		
≤ 50 vs > 50				
Gender	0.784	1.092 (0.581–2.054)		
Male vs female				
Tumor diameter (cm)	0.001**	2.999 (1.612–5.577)	0.228	1.536 (0.765–3.086)
≤ 4 vs > 4				
Tumor location	0.693	0.877 (0.457–1.681)		
Up + Middle vs Down				
Tumor differentiation	0.043*	1.895 (1.021–3.515)	0.353	1.361 (0.710–2.610)
High + Middle vs Low				
CEA (ng/ml)	0.135	1.755 (0.839–3.672)		
≤ 5 vs > 5				
CA 19-9 (U/ml)	0.845	0.926 (0.429–2.000)		
≤ 37 vs > 37				
HP infection	0.746	0.905 (0.494–1.658)		
− vs +				
T stage	0.002**	3.270 (1.561–6.849)	0.724	0.842 (0.325–2.184)
T1-2 vs T3-4				
N stage	< 0.0001****	7.548 (3.324–17.140)	0.017*	3.188 (1.235–8.230)
N0 vs N1-3				
HOXA-AS3 in tissues	< 0.0001****	7.462 (3.420–16.283)	0.005**	4.014 (1.534–10.508)
High vs Low				

*P < 0.05; **P < 0.01; ****P < 0.0001

### Knocking down HOXA-AS3 suppresses the proliferation, invasion, and migration of GC cells

**Fig. 2:**
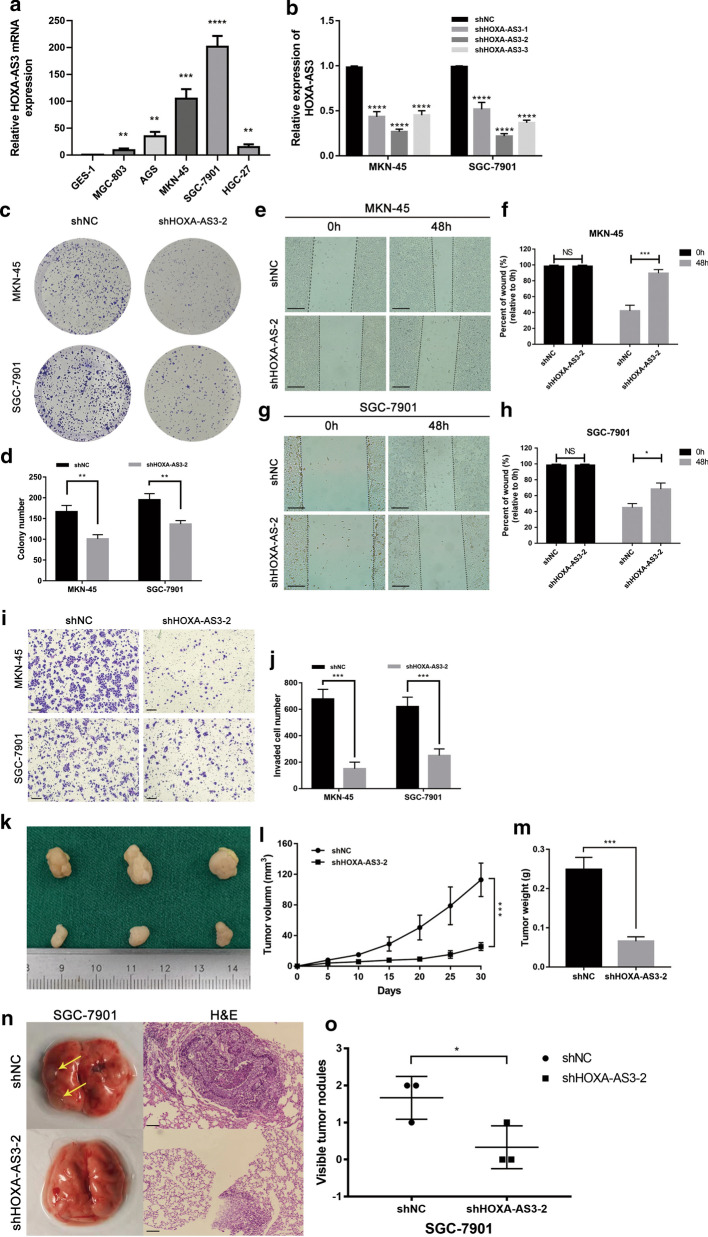
HOXA-AS3 knockdown suppresses GC cell proliferation, invasion, and migration. **a** HOXA-AS3 levels were compared in GC and control cell lines. **b** Relative HOXA-AS3 expression was evaluated in GC cells following transfection with three different shRNAs specific for this lncRNA. **c, d** The relative proliferation of GC cells following shHOXA-AS3 or shNC transfection was assessed via colony formation assay. **e**–**g** The impact of HOXA-AS3 knockdown on MKN-45 (**e**, **f**) and SGC-7901 (**g**, **h**) cellular migration was assessed via wound healing assay (scale bar, 100 μm). **i**,** j** Transwell assays were used to assess the proliferation and invasion of MKN-45 and SGC-7901 cells transfected as in c-d (scale bar, 100 μm). **k** Nude mice were subcutaneously implanted with SGC-7901 cells transfected with shHOXA-AS3 or shNC, with representative xenograft tumor images being shown. **l, m** Xenograft tumor weight and volume were compared in the two treatment groups. **n** HOXA-AS3 suppressed the in vivo metastasis of GC cells. Left: representative lung bright-field images, with lesions marked by arrows. Right: representative H&E-stained images of lung nodules (scale bar, 25 μm). **o** Numbers of metastases were quantified. Data are means ± SD from triplicate experiments. ^*^P < 0.05, ^**^P < 0.01, ^***^P < 0.001, ^****^P < 0.0001. *NS* no significance

We next determined that HOXA-AS3 was also overexpressed in GC cell lines relative to the control human GES-1 epithelial cells, with this upregulation being most pronounced in MKN-45 and SGC-7901 cells (*P* < 0.001 and *P* < 0.0001, Fig. 2a). We next conducted loss-of-function studies by knocking down HOXA-AS3 using an shRNA construct in MKN-45 and SGC-7901 cells (Fig. 2b). A subsequent colony formation assay revealed that GC cell proliferation was markedly impaired following HOXA-AS3 knockdown (Fig. 2c, d), and wound healing assays further revealed that knocking down this lncRNA impaired the migration of both of these cell lines relative to shNC transfection (Fig. 2e–h), with Transwell assays further confirming this result (Fig. 2i, j). Given the ability of HOXA-AS3 knockdown to compromise MKN45 and SGC7901 cell migration in vitro, we next generated SGC-7901 cells stably transduced with shHOXA-AS3 or shNC constructs, and then subcutaneously injected these into nude mice to generate a xenograft model system. Relative to shNC tumors, tumors in which HOXA-AS3 had been knocked down were significantly smaller (Fig. 2k–m). When these same cells were injected into mice via the tail vein to establish a model of lung metastasis, we similarly found that HOXA-AS3 knockdown was linked to a significant reduction in the number of pulmonary nodules relative to shNC control cells (Fig. 2n, o). As such, we concluded that HOXA-AS3 knockdown in vivo was sufficient to disrupt GC progression.

### HOXA-AS3 modulates NF-κB signaling to regulate GC cell biology

**Fig. 3:**
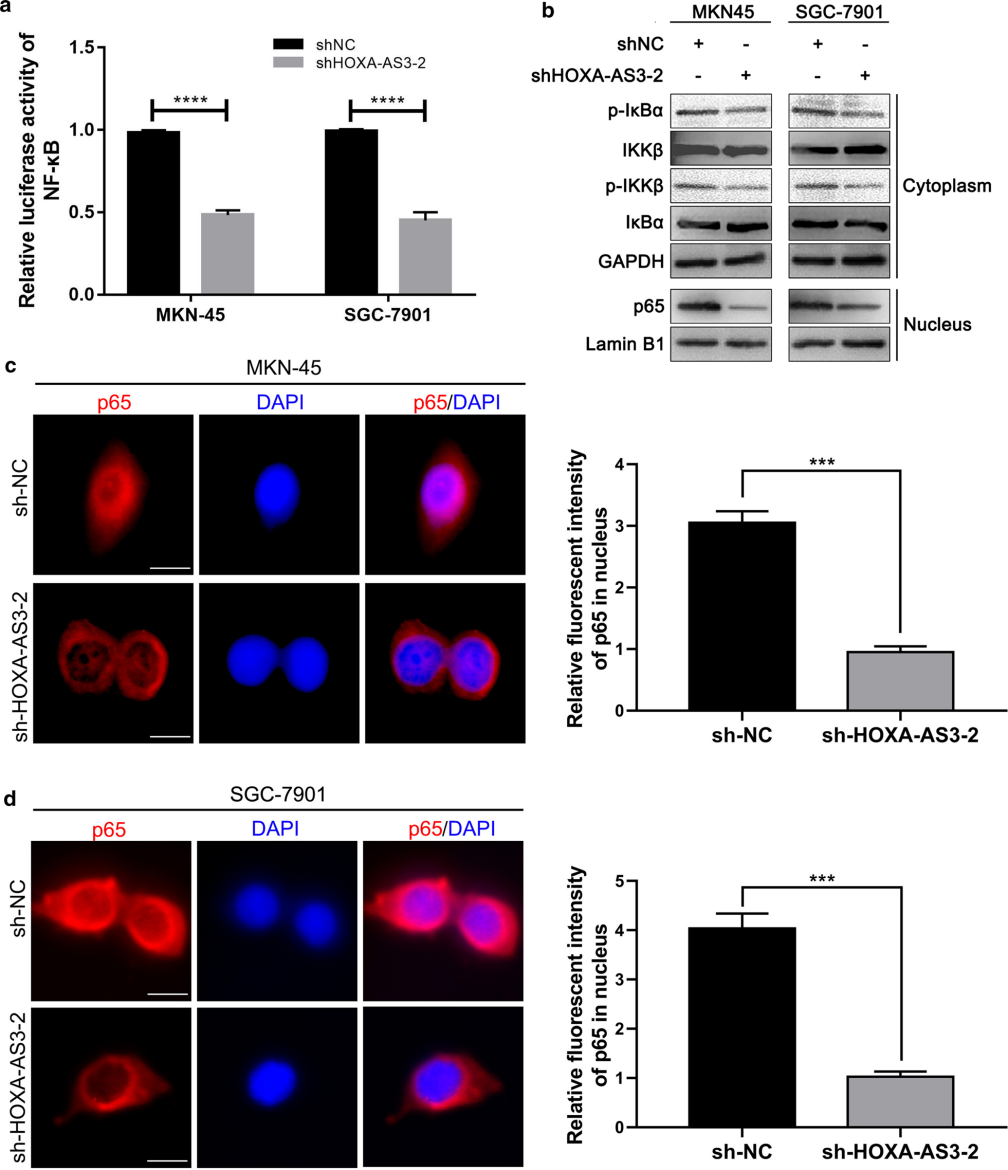
HOXA-AS3 knockdown suppresses NF-κB signaling in GC. **a** The impact of HOXA-AS3 knockdown on NF-κB luciferase reporter activity was analyzed in GC cells. **b** The effect of HOXA-AS3 knockdown on the expression of NF-κB-related signaling components in GC cell lines was evaluated via Western blotting, with GAPDH as a loading control. **c**,** d** The effect of HOXA-AS3 knockdown on the nuclear localization of p65 was assessed via immunofluorescent staining in MKN-45 (**c**) and SGC-7901 cells (**d**) (scale bar, 25 μm). Error bars: mean ± SD from triplicate experiments. ^****^P < 0.0001

In previous reports, HOXA-AS3 has been shown to positively regulate NF-κB signaling by interacting with this transcription factor [16], and abnormal NF-κB signaling can promote GC progression [17–19]. As such, we evaluated the ability of HOXA-AS3 to modulate NF-κB signaling in our GC model system. Through a luciferase reporter assay, we demonstrated that HOXA-AS3 knockdown suppressed NF-κB transcriptional activity (Fig. 3a). Consistent with this, Western blotting revealed that IKKβ and IκBα phosphorylation levels were decreased in cells in which this lncRNA had been knocked down relative to control cells in the shNC group for both tested GC cell lines (Fig. 3b). Immunofluorescence experiments also demonstrated that shHOXA-AS3 treatment was associated with a decrease in p65 nuclear translocation (Fig. 3c, d).

### HOXA-AS3 binds to miR-29a-3p in GC cells

**Fig. 4:**
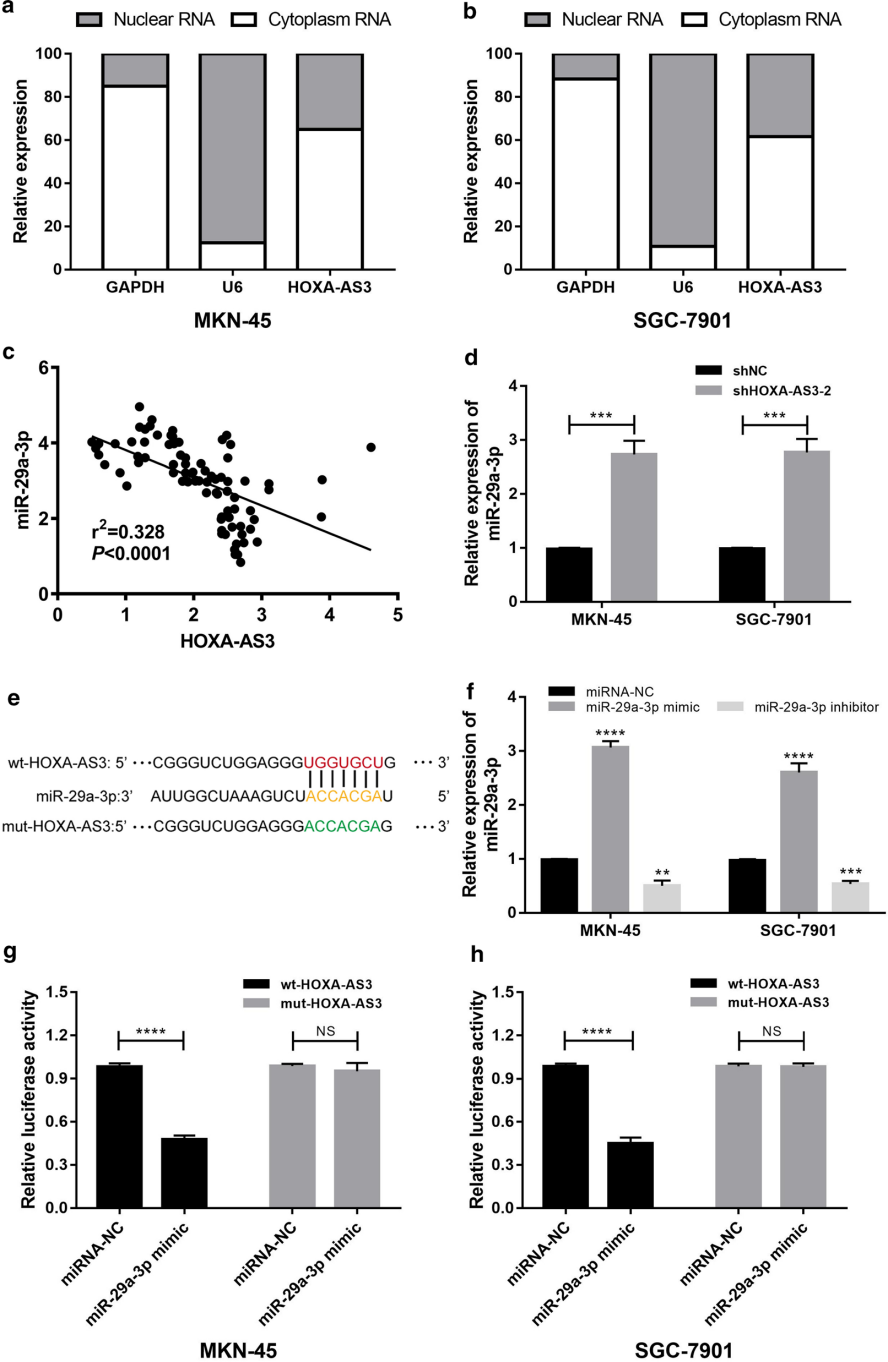
miR-29a-3p is a direct HOXA-AS3 target in GC. **a**,** b** Levels of HOXA-AS3 in the cytoplasm and nuclei of MKN-45 (**a**) and SGC-7901 (**b**) cells were measured via qPCR, with U6 and GAPDH as respective nuclear and cytoplasmic controls. **c** A negative correlation between HOXA-AS3 and miR-29a-3p expression was detected via qPCR in 82 GC patient tumor samples. **d** A qPCR approach was utilzied to measure miR-29a-3p levels in MKN-45 and SGC-7901 cells following HOXA-AS3 or control construct transfection. **e** Putative HOXA-AS3 sequence complementarity with miR-29a-3p, with mutated residues in reporter constructs being shown. **f** miR-29a-3p levels were quantified in GC cells following miR-29a-3p mimic or inhibitor transfection. **g**, **h** The impact of miR-29a-3p overexpression on WT or MUT HOXA-AS3 luciferase activity was quantified in MKN-45 (**h**) and SGC-7901 (**i**) cells. Error bars: mean ± SD from triplicate experiments. ^**^P < 0.01, ^***^P < 0.001, ^****^P < 0.0001. NS: no significance

We next conducted a subcellular fractionation experiment, which revealed higher levels of HOXA-AS3 in the cytoplasm relative to the nucleus (Fig. 4a, b). Such cytoplasmic localization is consistent with the ability of HOXA-AS3 to serve as a ceRNA for specific miRNAs, as has previously been shown to occur in the context of HCC, glioma, and atherosclerosis [9, 12, 20]. Using the Starbase v3.0 (http://starbase.sysu.edu.cn) platform [21], we identified three putative HOXA-AS3 targets in GC (miR-29a-3p, miR-29b-3p and miR-29c-3p). Of these three miRNAs, only low levels of miR-29a-3p expression were associated with poor GC patient prognosis in the TCGA database (*P* = 0.0412, Additional file 1: Fig. S1). We then assessed the expression of miR-29a-3p in GC cells and tissues via qPCR, revealing HOXA-AS3 and miR-29a-3p to be negatively correlated with one another in GC patient tumor samples (r^2^ = 0.328, *P* < 0.001, Fig. 4c). Knocking down HOXA-AS3 also resulted in miR-29a-3p upregulation in MKN-45 and SGC-7901 cells (Fig. 4d), consistent with these findings. We then conducted a luciferase reporter assay, which confirmed that miR-29a-3p mimic transfection was sufficient to suppress the activity of the HOXA‐AS3‐WT reporter construct, whereas miR-29a-3p inhibitor transfection had the opposite effect. In contrast, mimic and inhibitor transfection had no impact on HOXA‐AS3‐Mut luciferase activity (Fig. 4e–h). These findings thus provided clear evidence for the ability of HOXA-AS3 to target miR-29a-3p in GC.

### The HOXA-AS3/miR-29a-3p axis regulates LTβR expression in GC

**Fig. 5:**
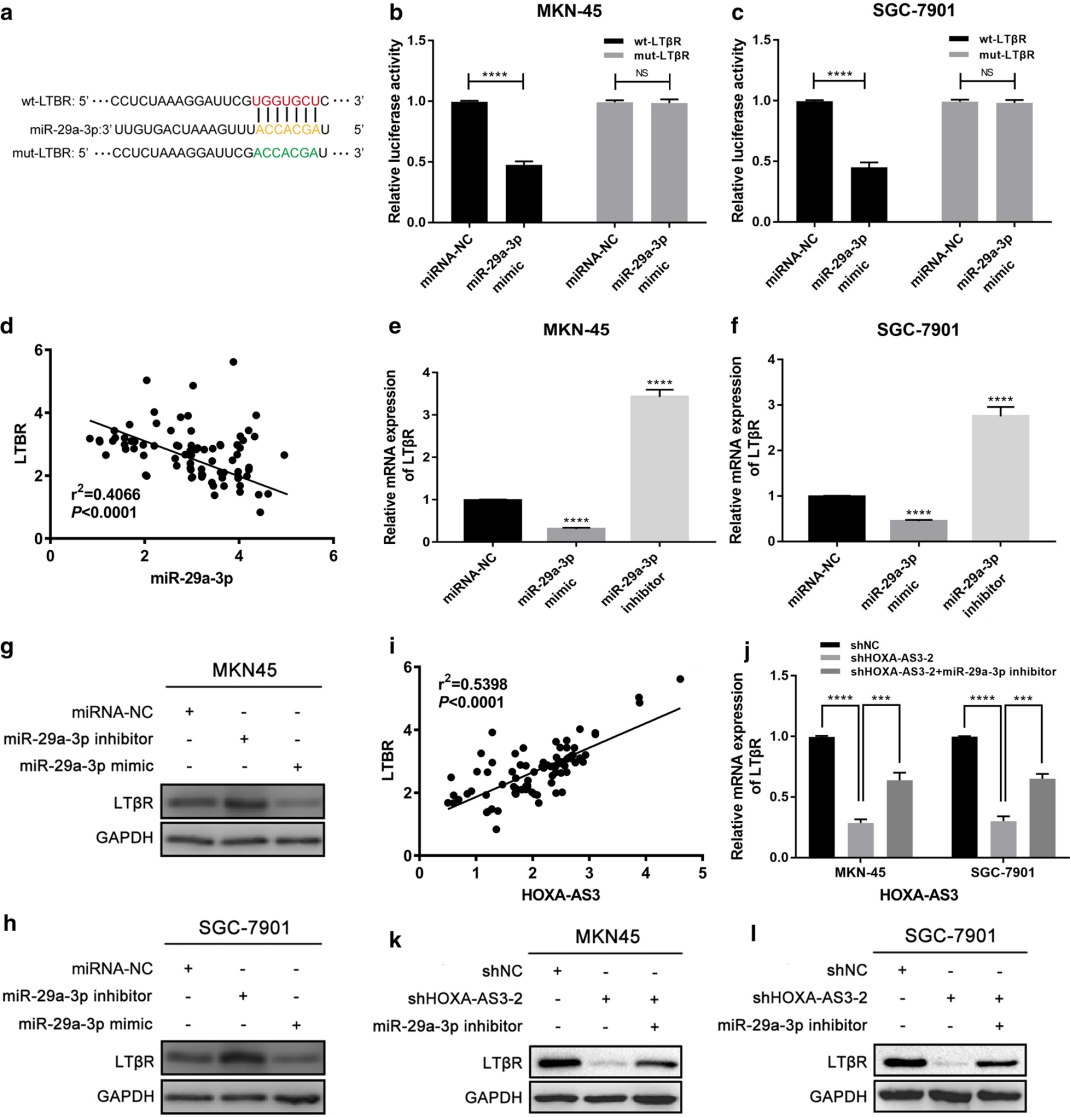
The HOXA-AS3/miR-29a-3p axis regulates LTβR expression in GC. **a** TargetScan was used to identify putative miR-29a-3p binding sites in the LTβR 3′-UTR, with mutated residues in reporter constructs being as indicated. **b**, **c** The impact of miR-29a-3p overexpression on WT and MUT LTβR luciferase reporter activity in MKN-45 (**b**) and SGC-7901 (**c**) cells was measured. **d** LTβR expression was negatively correlated with that of miR-29a-3p in 82 GC patient tumor samples. **e**, **f** Relative LTβR expression was evaluated via qPCR in MKN-45 (**e**) and SGC-7901 (**f**) cells after miR-29a-3p mimic or inhibitor transfection. **g**, **h** Western blotting was utilized to assess LTβR protein expression in MKN-45 (**g**) and SGC-7901 (**h**) cells following miR-29a-3p mimic or inhibitor transfection, with GAPDH as a loading control. **i** LTβR expression was positively correlated with that of HOXA-AS3 in 82 GC patient tumor samples, as assessed via qPCR.** j** Relative LTβR expression in MKN-45 (**e**) and SGC-7901 (**f**) cells following shHOXA-AS3-2 and/or miR-29a-3p inhibitor transfection. **k**, **l** LTβR protein levels were measured in GC cells transfected with shHOXA-AS3-2 and/or miR-29a-3p inhibitor via Western blotting, with GAPDH as a loading control. Error bars: mean ± SD from triplicate experiments. ^****^P < 0.0001. *NS* no significance

The MIRwalk, TargetScan, RNA22, and mirtarbase tools were next used to identify 187 putative miR-29a-3p target genes, including LTβR (Additional file 1: Fig. S2). We identified one predicted miR-29a-3p binding site within LTβR (Fig. 5a), which has previously been shown to mediate alternative NF-κB signaling in the context of *H. pylori-*induced gastric inflammation [22]. To validate this interaction between miR-29a-3p and LTβR in GC cells, we next performed a luciferase assay which confirmed that miR-29a-3p mimic co-transfection was sufficient to disrupt the activity of the WT but not the MUT LTβR luciferase reporter construct, whereas miR-29a-3p inhibitor cotransfection enhanced this activity (Fig. 5b, c). In addition, LTβR and miR-29a-3p levels were negatively correlated in 82 GC tissue samples (r^2^ = 0.4066, *P* < 0.05, Fig. 5d). Levels of LTβR mRNA and protein were decreased in GC cells transfected with a miR-29a-3p mimic, whereas miR-29a-3p inhibitor transfection had the opposite effect (Fig. 5e–h). These findings indicated that LTβR is a miR-29a-3p target in GC. As such, we next evaluated the ability of HOXA-AS3 expression to influence LTβR expression in these cells. A significantly positive correlation between LTβR and HOXA-AS3 expression in 82 GC tissues (r^2^ = 0.5398, *P* < 0.05, Fig. 5i). LTβR expression was confirmed to be reduced at the RNA and protein levels in GC cells transfected with the shHOXA-AS3-2 construct, while miR-29a-3p inhibitor transfection was sufficient to partially reverse the impact of HOXA-AS3 on the expression of LTβR (Fig. 5j–l). These findings indicated that HOXA‐AS3 can control the expression of LTβR by suppressing miR-29a-3p.

### The HOXA-AS3/miR-29a-3p/LTβR axis controls GC cell malignancy by regulating NF-κB signaling

**Fig. 6:**
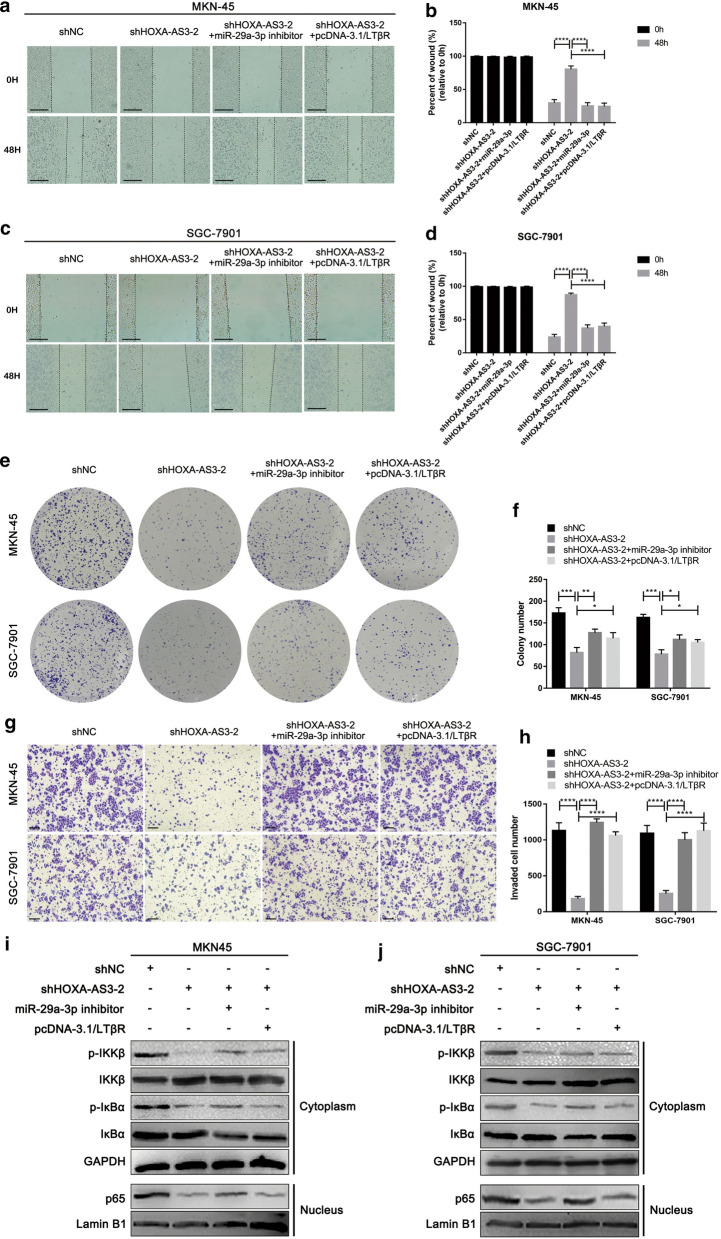
The HOXA-AS3/miR-29a-3p/LTβR axis controls NF-κB signaling and thereby regulates the proliferation, invasion, and migration of GC cells. **a**–**d** The migratory activity of MKN-45 (**a**–**b**) or SGC-7901 (**c**–**d**) cells in which HOXA-AS3 had been knocked down was assessed via wound healing assay following miR-29a-3p inhibitor or pcDNA-3.1/LTβR transfection. **e**, **f** Representative colony formation assay results for cells treated as in **a**–**d**. **g**, **h** Matrigel transwell assays were used to assess the invasive activity of cells treated as in **a**–**d**. **i**,** j** Western blotting was used to evaluate NF-κB signaling activity in cells treated as in **a**–**d**. GAPDH was utilized as a normalization control

Next, we conducted rescue experiments with the goal of establishing the relationship between miR-29a-3p, LTβR, and the ability of HOXA-AS3 to regulate GC cell proliferative and migratory characteristics. When cells were transfected with a miR-29a-3p inhibitor, this was sufficient to reverse the impact of HOXA-AS3 knockdown on GC cell proliferative and migratory activity, and LTβR overexpression had a comparable effect in these cells. Together, these data showed that miR-29a-3p/LTβR axis is a key mediator of the ability of HOXA-AS3 to regulate the malignant characteristics of GC cells (Fig. 6a–h).

As we demonstrated that HOXA-AS3 controls GC cell proliferation, migration, and invasion through the NF-κB signaling pathway, we next evaluated changes in NF-κB-related gene expression following GC cell transfection with sh-HOXA-AS3-2, pcDNA-3.1/LTBR, and miR-29a-3p inhibitor constructs. Western blotting revealed that miR-29a-3p inhibitor transfection or LTβR overexpression were sufficient to enhance IKKβ and IκBα phosphorylation and nuclear p65 localization in these GC cells (Fig. 6i, j). These results suggested that HOXA-AS3 can promote nuclear p65 accumulation by inhibiting miR-29a-3p and thereby enhancing the expression of LTβR. We thus speculate that this HOXA-AS3/miR-29a-3p/LTβR axis controls NF-κB signaling and thereby regulates GC progression.

## Discussion

Over 40% of global GC cases occur in China, with the majority of these patients being diagnosed when the disease is in a later stage, at which time curative surgical tumor resection is no longer an option [1, 2]. This delayed diagnosis, coupled with the high rates of GC tumor chemoresistance, results in a poor prognosis for the majority of these GC patients. As such, it is vital that novel diagnostic biomarkers and therapeutic targets associated with this cancer type be identified in order to provide a foundation for the development of novel personalized treatment strategies capable of markedly improving patient outcomes.

HOXA-AS3 is a lncRNA that was recently discovered and that was found to play a role in EZH2-dependent H3K27 trimethylation and consequent regulation of the ability of human bone marrow mesenchymal stem cells to undergo differentiation into osteoblasts or adipocytes [23]. In oncogenic contexts, HOXA-AS3 has also been shown to promote the proliferation, survival, and proliferation of glioma cells [8], while knocking down this lncRNA in lung adenocarcinoma was sufficient to impair the proliferative and invasive activity of these cells [10]. HOXA-AS3 has also been identified as a novel target in NSCLC patients owing to its ability to downregulate HOXA3 and to thereby modulate tumor cell resistance to cisplatin treatment [11]. However, no prior studies have explored the role of this lncRNA in GC. Herein, we determined that GC patient tumor tissues and cell lines exhibit HOXA‐AS3 upregulation that was correlated with tumor size, *H. pylori* infection status, T stage, and N stage. Consistent with findings in glioma and HCC patients [8, 9], this elevated HOXA-AS3 expression was an independent predictor of poor GC patient prognosis. When we knocked down this lncRNA in GC cells, we found that this was sufficient to compromise their growth and metastatic potential in vitro and in vivo*.* Overall, these data suggested that HOXA-AS3 functions to promote GC development and progression, making it a potentially viable biomarker for the evaluation of GC patient disease status and prognosis. However, studies with a larger sample size studies are needed to validate the clinical significance of this biomarker.

NF-κB signaling is a central mediator of GC progression [24]. HOXA-AS3 is able to colocalize with NF-κB in the promoter region of certain genes, and can directly interact with this transcription factor in order to enhance its activation by regulating IκBα expression and p65 subunit K310 acetylation status [16]. As such, we speculated that HOX-AS3 may drive GC progression via the activation of NF-κB. Our experiments confirmed this hypothesis, as knocking down HOXA-AS3 was sufficient to suppress NF-κB, whereas activating NF-κB reversed the negative impacts of HOXA-AS3 knockdown on GC cell proliferative and migratory activity.

There is clear evidence that HOXA-AS3 can function as a ceRNA in the context of HCC, glioma, and atherosclerosis [9, 12, 20]. In HCC, this lncRNA controls the miR-29c/BMP1 axis and thereby promotes tumor cell proliferation, EMT, and metastasis via activating MEK/ERK signaling [9]. As such, we hypothesized that HOXA-AS3 may similarly control NF-κB signaling activity by functioning as a ceRNA in GC. To that end, we identified putative miRNA targets of HOXA-AS3 in GC via the use of a predictive bioinformatics program, leading to the identification of miR-29c and miR‐29a-3p as complementary binding targets. Given that miR‐29a-3p had previously been shown to be expressed at low levels in GC and to be correlated with poor GC patient prognosis [25, 26], we examined this miRNA target in-depth and confirmed that it was able to directly bind to HOXA‐AS3, which negatively regulated its expression and function. We further identified LTβR as a downstream miR‐29a-3p target, such that HOXA‐AS3 was able to indirectly control the expression of this gene by modulating miR‐29a-3p activity. Prior studies have demonstrated that LTβR can induce non-classical NF-κB signaling in the context of *H. pylori* infection, intestinal metaplasia, and atypical hyperplasia, the exact mechanistic role of this gene in GC remains to be studied in detail [22, 27]. Even so, we were able to demonstrate through the use of luciferase reporter assays that the HOXA-AS3 knockdown-dependent inhibition of NF-κB activation was reversed when GC cells overexpressed LTβR or were transfected with a miR-29a-3p inhibitor, and Western blotting further confirmed that these manipulations were sufficient to reverse the HOXA-AS3 knockdown-dependent inhibition of IKKβ, IκBα phosphorylation, and nuclear P65 expression in GC cells. As such, our data provide clear evidence that the HOXA-AS3/miR-29a-3p/LTβR signaling axis plays a key role in the regulation of GC development owing to its ability to modulate NF-κB signaling. However, available data on this subject were limited. It remains unclear whether certain transcription factors also regulate HOXA-AS3 in GC. Besides, considering previous study in HCC [9], whether this lncRNA might regulate other signaling pathways to exert its effect on GC development and progression remains mysterious. Therefore, these issues are needed to further investigate in our future studies.

## Conclusions

In summary, HOXA-AS3 is a lncRNA that is expressed at high levels in GC wherein it is correlated with T stage, N stage, tumor size, *H. pylori* infection status, and a poor patient prognosis. We also observed decreased miR-29a-3p expression and enhanced LTβR expression in GC, and determined that this HOXA-AS3 was able to negatively regulate miR-29a-3p and to thereby promote LTβR upregulation, thus driving GC progression via NF-κB pathway activation. Overall, our data offer new insights into the pathogenesis of GC and highlight the potential relevance of HOXA-AS3 as a diagnostic or therapeutic target in this cancer type.

## Supplementary Information

**Additional file 1: Figure S1.** GC patients with high and low levels of miR-29a-3p expression (n=200 each) were compared to assess their relative OS by OncoLnc. **Figure S2.** Venn plot was used to illustrate 187 potential target genes of miR-29a-3p by RNA22, mirtarbase, targetscan and MIRwalk.

## Data Availability

The datasets used and/or analyzed during the current study are available from the corresponding author on reasonable request.

## Abbreviations

ceRNA: Competing endogenous RNA
DFS: Disease-free survival
FBS: Fetal bovine serum
GC: Gastric cancer
H&E: Hematoxylin and eosin
HCC: Hepatocellular carcinoma
HOXA-AS3: HOXA cluster antisense RNA 3
lncRNAs: Long non-coding RNAs
miRNA: MicroRNA
OS: Overall survival
