# Supplementary material for: LncRNA HOXA-AS3 promotes gastric cancer progression by regulating miR-29a-3p/LTβR and activating NF-κB signaling
Source: Cancer Cell Int. 2021 Feb 18;21:118. doi: 10.1186/s12935-021-01827-w (PMC7890634; doi:10.1186/s12935-021-01827-w)

**Supplementary figure ledgends**

FigS.1

GC patients with high and low levels of miR-29a-3p expression (n=200 each) were compared to assess their relative OS by OncoLnc.

FigS.2

Venn plot was used to illustrate 187 potential target genes of miR-29a-3p by RNA22, mirtarbase, targetscan and MIRwalk.


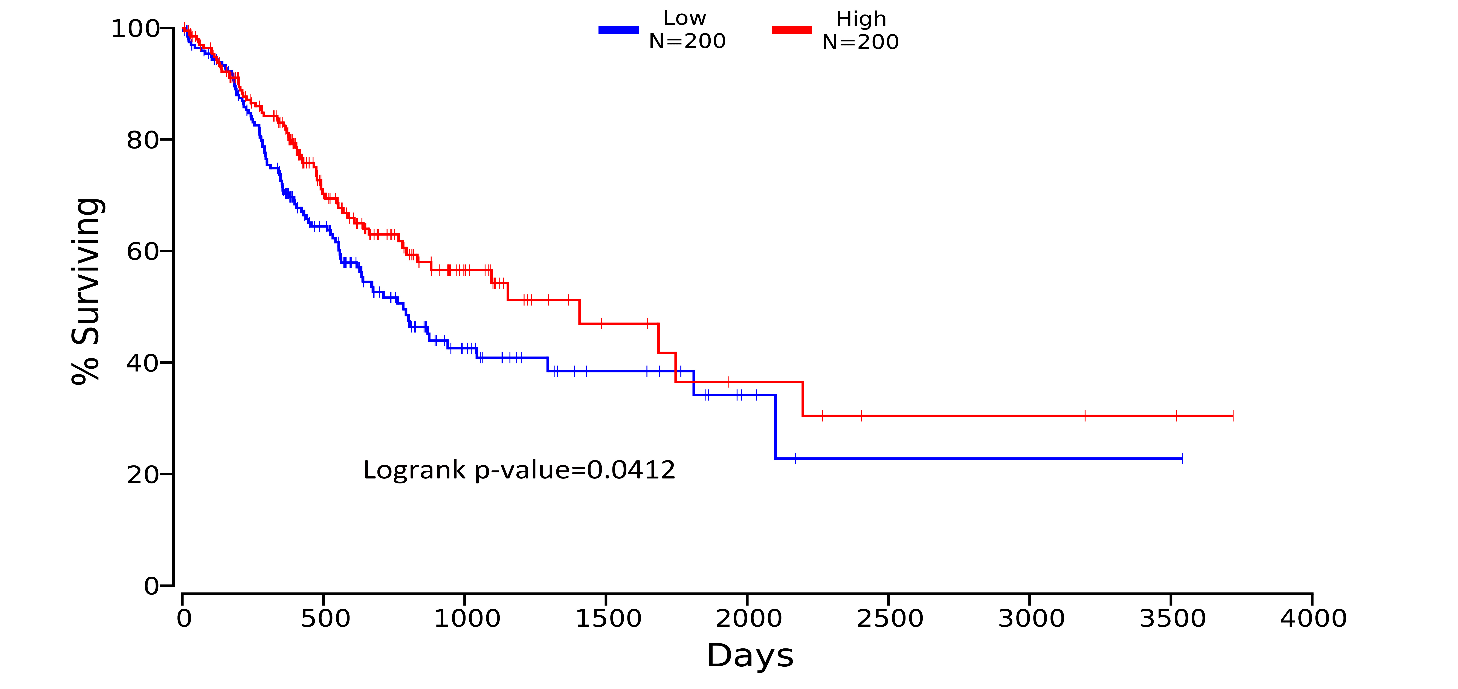


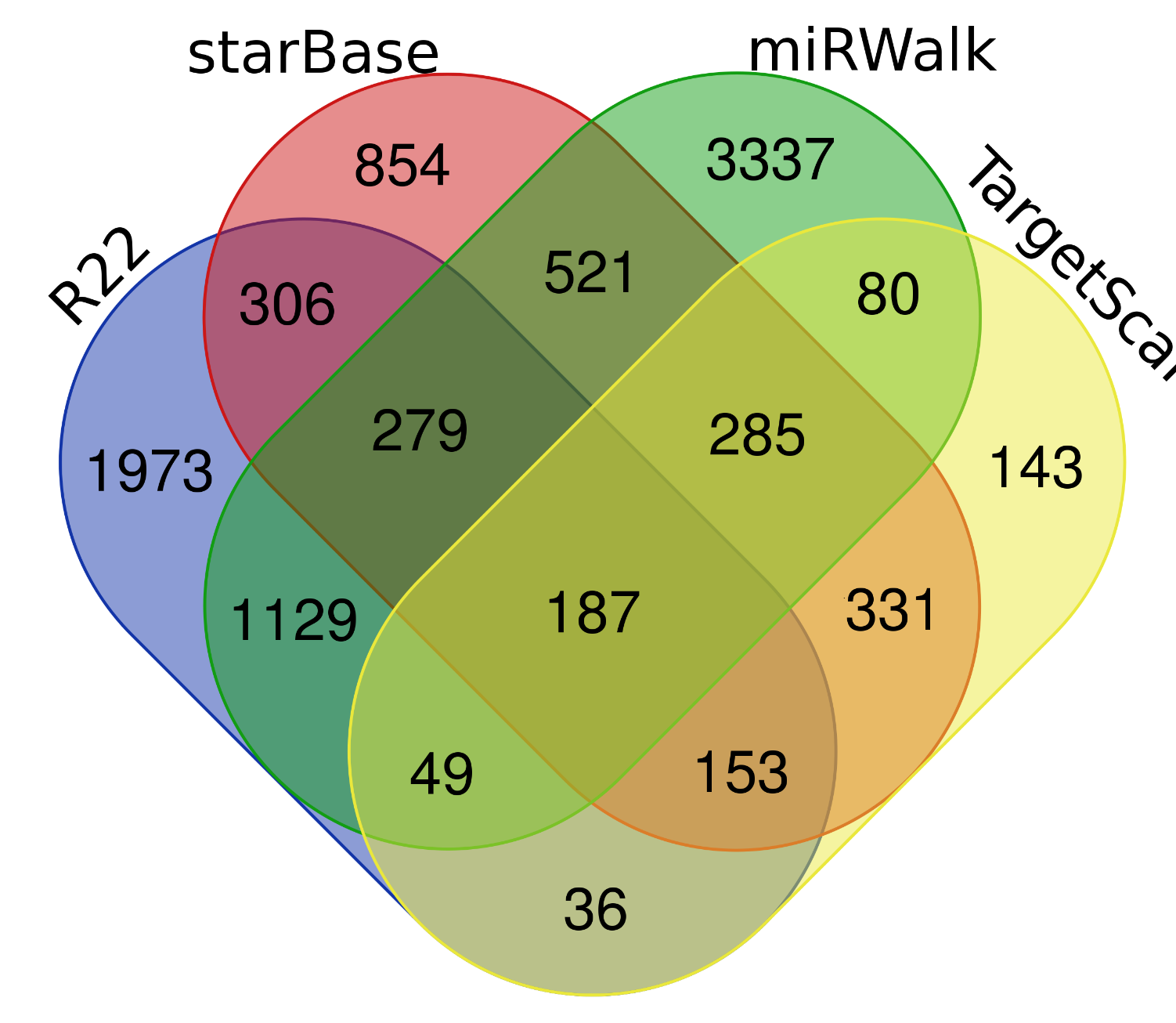

Supplement: Supplementary file 1 — Additional file 1: Figure S1. GC patients with high and low levels of miR-29a-3p expression (n=200 each) were compared to assess their relative OS by OncoLnc. Figure S2. Venn plot was used to illustrate 187 potential target genes of miR-29a-3p by RNA22, mirtarbase, targetscan and MIRwalk. [file 12935_2021_1827_MOESM1_ESM.docx]
